# The effect of premedication with butorphanol or methadone on the ease of duodenal intubation

**DOI:** 10.18849/ve.v10i3.715

**Published:** 2025-09-04

**Authors:** Charlotte Swanson+

**Keywords:** ANAESTHESIA, BUTORPHANOL, CANINE, DOGS, DUODENAL INTUBATION, ENDOSCOPY, METHADONE, PREMEDICATION, UPPER GASTROINTESTINAL

## Abstract

**PICO question:**

In adult dogs undergoing upper gastrointestinal (GI) endoscopy, does premedication with butorphanol compared to premedication with methadone make duodenal intubation easier?

**Clinical bottom line:**

**Category of research:**

Treatment.

**Number and type of study designs reviewed:**

Two prospective, randomised, blinded, clinical trials.

**Strength of evidence:**

Weak.

**Outcomes reported:**

The evidence from the two studies is contradictory. One reports that premedication with butorphanol is associated with significantly quicker and easier duodenal intubation compared to methadone. The other reports that methadone is non-inferior to butorphanol for ease of duodenal intubation and did not identify a significant difference in speed or ease of duodenal intubation between premedications. Direct comparison of the studies is limited by differing anaesthetic protocols and variation between the scoring systems for ease of intubation.

**Conclusion:**

There is insufficient evidence to support the use of butorphanol over methadone as a premedication in dogs undergoing upper GI endoscopy requiring duodenal intubation. The process of duodenal intubation may be quicker and easier with butorphanol versus methadone, but duodenal intubation can be successful with both opioids, and there is no difference in the requirement for rescue analgesia between the two drugs.

## How to apply this evidence in practice

The application of evidence into practice should take into account multiple factors, not limited to: individual clinical expertise, patient’s circumstances and owners’ values, country, location or clinic where you work, the individual case in front of you, the availability of therapies and resources.

Knowledge Summaries are a resource to help reinforce or inform decision-making. They do not override the responsibility or judgement of the practitioner to do what is best for the animal in their care.

## Clinical scenario

A 3-year-old female neutered Labrador presents for upper gastrointestinal endoscopy to investigate chronic vomiting. The procedure requires general anaesthesia. Will the duodenal intubation be easier if butorphanol is used for premedication, or if methadone is given instead?

## The evidence

Two studies relevant to the PICO were reviewed (McFadzean et al., 2017; Salla et al., 2020). Both were prospective, randomised, blinded clinical trials. Overall, the results were contradictory, and the strength of the evidence is weak due to differences in study design. The use of a different version of an unvalidated scoring system with real-time single person analysis in each study, and the addition of acepromazine to the premedication protocol in one of the studies, results in the evidence being insufficient to support the use of butorphanol over methadone.

## Summary of the evidence

**McFadzean et al. (2017):** Effect of premedication with butorphanol or methadone on ease of endoscopic duodenal intubation in dogs **Aim: **To evaluate the effect of premedication with butorphanol or methadone on ease of endoscopic duodenal intubation.

Population:	Client-owned dogs scheduled for upper gastrointestinal endoscopy at the School of Veterinary Sciences, University of Bristol, United Kingdom.
Sample size:	20 dogs.
Intervention details:	Dogs were assigned to one of the two premedication groups using an online randomisation programme. Butorphanol 0.4 mg/kg intravenously (IV) (10 dogs). Methadone 0.3 mg/kg IV (10 dogs). Premedication diluted to a total volume of 0.05 ml/kg with sterile water for injection and administered IV. Each premedication was prepared by a second anaesthetist so the anaesthetist performing the study was blinded to the group allocations. 20 minutes after premedication: induction of anaesthesia with propofol given to allow tracheal intubation. Maintenance of anaesthesia with isoflurane at a concentration giving adequate depth of anaesthesia. Initiation of upper gastrointestinal tract endoscopy. Administration of a bolus of propofol 1 mg/kg IV if purposeful movement or swallowing occurred in response to passage of the endoscope into the stomach or through the pyloric sphincter. Increase in inspired isoflurane concentration by 0.25% if an increase in heart rate, respiratory rate or mean arterial pressure of > 20% was observed. Advancement of the endoscope into the duodenum after visualisation of the pylorus was achieved.
Study design:	Prospective, randomised, blinded clinical trial.
Outcome Studied:	Time from visualisation of the pylorus to advancing the endoscope through the pyloric sphincter into the duodenum. Ease of passing the endoscope through the pyloric sphincter, graded by a blinded observer in real-time using a 4-point scale (1 = immediate entry with minimal manoeuvering, 4 = no entry after 2 minutes).
Main Findings (relevant to PICO question):	Median time taken to pass the endoscope through the pyloric sphincter was significantly shorter in the butorphanol group (65 seconds) compared to methadone (120 seconds) (P = 0.028). Spontaneous opening of the pyloric sphincter was more common in the butorphanol group (7/10), compared to 2/10 in the methadone group, (P = 0.035). Duodenal intubation, rated using the 4-point scale above, was significantly easier for butorphanol (3 ± 1) vs methadone (4 ± _­_1) (P = 0.035).
Limitations:	The study population included a wide range of patient weights, but the size of the endoscope(s) used for the procedures have not been described. There is potential for a wide variation in the relative size of endoscope to patient. 7 different endoscopists carried out the procedure across 20 dogs. The experience levels of the endoscopists or their distribution between the two intervention groups is not described. The study did not use equipotent doses of butorphanol and methadone. The assessment scale classifies the highest score as ‘no entry after 2 minutes’. The paper does not mention whether duodenal intubation was eventually achieved in all cases where the 2-minute timeframe was exceeded. The ease of duodenal intubation was assessed visually in real time by a blinded observer. It is not explicitly specified whether this was the same person for all cases. The scoring system used to score the ease of duodenal intubation is not validated. The impact of a propofol bolus or increasing the isoflurane concentration on the ease of duodenal intubation has not been considered.

**Salla et al. (2020):** Comparison of the effects of methadone and butorphanol combined with acepromazine for canine gastroduodenoscopy **Aim: **To evaluate the feasibility of gastroduodenoscopy in dogs premedicated with butorphanol or methadone in combination with acepromazine.

Population:	Client-owned dogs scheduled for gastroduodenoscopy at the Department of Small Animal Medicine, University of Helsinki, Finland. Medium- to large-sized dogs with American Society of Anaesthesiologists (ASA) status scores of I–II.
Sample size:	40 dogs (3 dogs excluded from analysis as a full stomach precluded gastroduodenoscopy).
Intervention details:	Dogs were assigned to one of two groups using block randomisation with a 1:1 ratio. Butorphanol 0.3 mg/kg intramuscularly (IM) with 0.02 mg/kg acepromazine (20 dogs). Methadone 0.2 mg/kg IM with 0.02 mg/kg acepromazine (20 dogs). Each premedication was mixed in a single syringe and covered in tape by a third person, to blind the anaesthetist and endoscopist. IV catheter placement 20 minutes after administration of the premedication. Induction of general anaesthesia with 1 mg/kg ketamine IV followed by propofol IV (starting at 2 mg/kg) to allow tracheal intubation. Maintenance of anaesthesia using sevoflurane with an end-tidal concentration of 2.3%. Endoscopy was carried out by the same clinician. Administration of 3 mcg/kg fentanyl IV if two of the heart rate, respiratory rate or mean arterial pressure increased by over 20% from baseline during the procedure. Advancement of the endoscope into the duodenum after visualisation of the pylorus.
Study design:	Prospective, randomised, double-blinded, clinical, non-inferiority trial.
Outcome Studied:	Time taken from visualisation of the pyloric sphincter to achieving a tubular image of the proximal duodenum. Score for pyloric intubation using a 4-point scale, graded in real time by the endoscopist (1 = no resistance to pass through the pylorus, 4 = duodenum not reached). General feasibility of the gastroduodenoscopy, graded by the endoscopist at the end of the procedure using a visual analogue scale (VAS) from 0 to 100 (where 0 is the most feasible gastroduodenoscopy and 100 is an unsuccessful procedure).
Main Findings (relevant to PICO question):	Data from 37 dogs was analysed (20/20 given butorphanol, 17/20 given methadone). Three dogs were excluded from analysis as gastroduodenoscopy was aborted due to a full stomach. Duodenal intubation was achieved in all dogs. No difference in the time to reach the duodenum between intervention groups: butorphanol group 32.5 ±4 seconds, methadone 47.8 ± 32.9 seconds (P = 0.168). No difference in pyloric intubation scores between groups (P = 0.08). No significant difference in VAS score for feasibility of gastroduodenoscopy between methadone and butorphanol groups, based on a margin for non-inferiority of -10 and a confidence interval of 95% (P = 0.25)
Limitations:	Justification for the chosen doses of butorphanol and methadone was not given. Administration of acepromazine in the premedication may have facilitated passage of the endoscope into the duodenum, which may have reduced the ability of the study to differentiate between the effects of butorphanol and methadone on duodenal intubation. Fentanyl was used for intraoperative rescue analgesia in the study protocol. The potential impact of the mu opioid receptor agonism of fentanyl at the pyloric sphincter on duodenal intubation was not considered in the discussion. Ease of duodenal intubation, including resistance to passage of the endoscope through the pyloric sphincter, was assessed in real time by a single blinded endoscopist. Analysis of video recordings of the endoscopy by multiple observers could have reduced some of the subjectivity of the assessment but would not have enabled assessment of the resistance to passage of the endoscope. The scoring system used to score the ease of duodenal intubation is not validated.

## Appraisal, application and reflection

Two original research papers were identified which addressed the PICO question (McFadzean et al., 2017; Salla et al., 2020). Both were prospective, randomised, blinded, clinical trials comparing the effect of butorphanol with methadone on the ease of duodenal intubation, and appropriate power calculations were provided for this analysis. The findings of the two papers contradict each other, with the conflicting results likely arising from the addition of acepromazine to the premedication in one of the studies, and overall they provide weak clinical evidence. McFadzean et al. (2017) found that duodenal intubation was easier after premedication with butorphanol than with methadone, and the time taken was significantly shorter. Salla et al. (2020) found that there was no significant difference in the time taken for duodenal intubation between dogs that received butorphanol and acepromazine as a premedication, compared to methadone and acepromazine. This study also found no significant difference in the ease of duodenal intubation between the two groups. Analysis of visual analogue scores found methadone was non-inferior to butorphanol in premedication for duodenal intubation when administered with acepromazine. Direct comparison between the study outcomes is limited by key differences in the study protocols.

The two studies used different doses of butorphanol and methadone in their protocols. McFadzean et al. (2017) note that the opioid doses used, butorphanol 0.4 mg/kg IV and methadone 0.3 mg/kg intravenously (IV), had equal efficacy as premedicants as they did not produce significantly different sedation scores. However, the correlation between sedative effect and effect on the pyloric sphincter is not known. Similarly, Salla et al. (2020) use lower doses of both opioids in combination with acepromazine, without significant difference in sedation score, but with unknown relative effects on the pyloric sphincter. Importantly, both studies use clinically relevant doses of butorphanol and methadone.

Coadministration of the opioid with acepromazine in Salla et al. (2020) may have reduced the ability to detect a difference between the two groups by facilitating passage of the endoscope into the duodenum. This effect is hypothesised to occur due to both the dopamine D2 receptor and alpha-1 adrenoceptor antagonism effects of acepromazine reducing motor activity at the gastroduodenal junction (Salla et al., 2020). Donaldson et al. (1993), however, found that acepromazine 0.05 mg/kg IM as a premedication did not produce a significant difference in the ease of duodenal intubation when compared with 0.9% saline, so the clinical effect of the 0.02 mg/kg given IM in Salla et al. (2020) may not be clinically significant.

The use of fentanyl as intraoperative rescue analgesia in the study protocol in Salla et al. (2020) may also have reduced the ability to detect a difference between premedication protocols. Fentanyl is a mu opioid receptor agonist so, similarly to methadone, is likely to act on the pyloric sphincter. There was no significant difference between the number of dogs requiring fentanyl between groups, but dogs in both groups were given fentanyl during the gastroduodenoscopy itself. It was given in 10/20 dogs premedicated with butorphanol and acepromazine and 6/17 dogs given methadone and acepromazine. Fentanyl was given during the duodenoscopy itself in 5/10 dogs given butorphanol and acepromazine that required rescue analgesia, and 2/6 dogs given methadone and acepromazine. Fentanyl has both mu and delta opioid agonist effects, but its effect on duodenal intubation has not been investigated.

It is also important to note that while McFadzean et al. (2017) identified a significant difference in the ease of duodenal intubation between butorphanol and methadone premedication groups, the study involved seven different endoscopists and a wide range of patient sizes (20.0 ± 12.9 kg (butorphanol group) and 14.2 ± 8.4 kg (methadone group)). Endoscopist experience level can significantly affect the time taken for duodenal intubation (Matz et al., 1991). The impact of different endoscopists in McFadzean et al. (2017) was not controlled for prospectively or using a regression analysis so its impact is unclear. The paper states in the discussion that endoscopist experience was not significantly correlated with the time taken for duodenal intubation or the ease of passing the endoscope through the pyloric sphincter, but evaluation of the evidence is limited as neither the data nor the statistical analysis are presented. The size of endoscope(s) used is not described, so the relative size of endoscope to patient is unknown, but a larger endoscope relative to patient size may result in more challenging duodenal intubation. In Salla et al. (2020), the same endoscopist was used for all procedures, there was a narrower range of patient weights (25.9 ± 6.0 kg (butorphanol and acepromazine) and 25.4 ± 5.3 kg (methadone and acepromazine)), and in all but one dog the same size endoscope was used (12.8 mm in 36/37 dogs, 9.9 mm in 1/37 dogs), giving less variability.

Both studies used a scoring system based on Matz et al. (1991) to score the ease of duodenal intubation. This system is not validated, and it was modified in Salla et al. (2020) from the original version adopted by McFadzean et al. (2017). McFadzean et al. (2017) used visual assessment of the ease of manoeuvering the endoscope in real time by an external observer, whilst Salla et al. (2020) used assessment of the force required for and the ease of duodenal intubation by the endoscopist themselves. There may be a difference in the reliability or validity of these approaches, which in turn affects the reliability of the results.  Additionally, McFadzean et al. (2017) defined score 4 as ‘no entry [into the duodenum] after 2 minutes’, compared to ‘duodenum not reached’ in Salla et al. (2020). As the duodenal intubation was successful in all dogs in Salla et al. (2020), the range of scores recorded was 1–3 rather than 1–4, which may have reduced the ability to detect a significant difference between protocols.

In both studies the ease of duodenal intubation was assessed in real time by a single observer (McFadzean et al., 2017) or endoscopist (Salla et al., 2020) per case. Modern endoscopy equipment enables video recording of images. Analysis of video recordings by multiple observers may have reduced the impact of subjectivity on the assessment but would not have enabled assessment of the resistance to passage of the endoscope in Salla et al. (2020) so a combination of real-time and recorded assessment may have been appropriate.

Overall, there is insufficient evidence from these two papers to draw a conclusion on whether butorphanol or methadone premedication better aids duodenal intubation during upper gastrointestinal endoscopy, particularly if used in combination with other sedative drugs. Duodenal intubation was achieved in less than 2 minutes in all dogs in one study (Salla et al., 2020), and 14/20 (70%) of dogs in the other (McFadzean et al., 2017). Additionally, neither paper reports a significant difference in requirement for rescue analgesia or anaesthesia between groups. Although neither paper was powered for this analysis, it suggests that analgesia requirements also fail to provide a justification for choosing one of butorphanol and methadone over the other.

## Methodology

**Search Strategy:** 

Databases searched and dates covered:	CAB Abstracts on the OVID platform 1973 to 2024 Week 20 PubMed accessed via the NCBI website 1954 to May 2024
Search strategy:	CAB Abstracts: (dog or dogs or canine) (butorphanol or methadone or opioid) (duodenum or duodenal) (endoscopy or intubation) PubMed: ((((dog OR dogs OR canine)) AND ((butorphanol OR methadone OR opioid))) AND ((duodenum OR duodenal))) AND ((endoscopy OR intubation))
Dates searches performed:	15 May 2024

**Exclusion / Inclusion Criteria:** 

Exclusion:	Papers not relevant to the PICO question.
Inclusion:	Papers relevant to the PICO question.

**Search Outcome:** 

Database	Number of results	Excluded – not relevant to the PICO question	Total relevant papers
CAB Abstracts	4	4	2
PubMed	4	4	2
Total relevant papers when duplicates removed			2

## Acknowledgements

Thank you to Sean Langton for reviewing the manuscript prior to submission.

## ORCiD

Charlotte Swanson: https://orcid.org/0009-0000-3472-9527

## Conflict of Interest

The author declares no conflicts of interest.
